# Identifying symptoms of ovarian cancer: a qualitative and quantitative study

**DOI:** 10.1111/j.1471-0528.2008.01772.x

**Published:** 2008-07

**Authors:** CR Bankhead, C Collins, H Stokes-Lampard, P Rose, S Wilson, A Clements, D Mant, ST Kehoe, J Austoker

**Affiliations:** a Cancer Research UK Primary Care Education Research Group, University of OxfordHeadington, Oxford, UK; b Department of Obstetrics and Gynaecology, University of BirminghamEdgbaston, Birmingham, UK; c Department of Primary Care, University of BirminghamEdgbaston, Birmingham, UK; d Department of Primary Care, University of OxfordOxford, UK; e Nuffield Academic Department of Obstetrics & Gynaecology, University of Oxford, The Women's Centre, John Radcliffe HospitalHeadington, Oxford, UK

**Keywords:** Diagnosis, mixed methods, ovarian cancer, referral, symptoms

## Abstract

**Introduction:**

Symptoms of ovarian cancer are often vague and consequently a high proportion of women with ovarian cancer are not referred to the appropriate clinic.

**Objective:**

To identify diagnostic factors for ovarian cancer.

**Design:**

A qualitative and quantitative study.

**Setting:**

Four UK hospitals.

**Sample:**

One hundred and twenty-four women referred to hospital with suspected ovarian malignancy.

**Methods:**

Women were interviewed prior to diagnosis (*n* = 63), or soon after. A thematic analysis was conducted. Emergent symptoms were quantitatively analysed to identify distinguishing features of ovarian cancer.

**Main outcomes:**

Symptoms in women with and without ovarian cancer.

**Results:**

Diagnoses comprised 44 malignancies, 59 benign gynaecological pathologies and 21 normal findings. Of the malignancies, 25 women had stage III or more disease, with an average age of 59 years. The benign/normal cohort was significantly younger (48 years). Multivariate analysis revealed persistent abdominal distension (OR 5.2, 95% CI 1.3–20.5), postmenopausal bleeding (OR 9.2, 95% CI 1.1–76.1), appetite loss (OR 3.2, 95% CI 1.1–9.2), early satiety (OR 5.0, 95% CI 1.6–15.7) and progressive symptoms (OR 3.6, 95% CI 1.3–9.8) as independent, statistically significant variables associated with ovarian cancer. Fluctuating distension was not associated with ovarian cancer (OR 0.4, 95% CI 0–4.1). Women frequently used the term bloating, but this represented two distinct events: persistent abdominal distension and fluctuating distension/discomfort.

**Conclusions:**

Ovarian cancer is not a silent killer. Clinicians should distinguish between persistent and fluctuating distension. Recognition of the significance of symptoms described by women could lead to earlier and more appropriate referral.

*Please cite this paper as:* Bankhead C, Collins C, Stokes-Lampard H, Rose P, Wilson S, Clements A, Mant D, Kehoe S, Austoker J. Identifying symptoms of ovarian cancer: a qualitative and quantitative study. BJOG 2008;115:1008–1014.

## Introduction

Ovarian cancer is the most common gynaecological cancer in UK women.^1^ Each year, around 7000 women in the UK are diagnosed with ovarian cancer, and approximately 4400 women die from it.^2^ One of the most important prognostic factors is the stage at diagnosis. Women with early-stage disease have 5-year survival rates in excess of 70%, whereas that for advanced disease is around 15%.^2^ The majority of women are diagnosed at a late stage, and therefore, overall 5-year survival rates are correspondingly low at 30–40%.^2^

The presenting symptoms of ovarian cancer are not specific and are often accepted by women as normal changes associated with ageing, menopause and previous pregnancies.^3^ As a result, ovarian cancer is often referred to as the ‘silent killer’, and it is commonly believed that no symptoms are evident in early disease. Furthermore, referral decisions for GPs are frequently difficult due to the fact that the presenting symptoms for ovarian cancer are similar to those for gastrointestinal disease.^4^ Women often follow convoluted referral pathways before being correctly diagnosed, with 50% of women not being referred directly to gynaecological cancer clinics. This is due to both women and GPs failing to recognise the presenting symptoms of ovarian cancer.^5^,^6^

Given the relationship between stage at diagnosis and survival, there is an increasing emphasis on the need to develop effective strategies for earlier diagnosis, including the identification of symptoms predictive of ovarian cancer. Several previous research studies have compared symptoms among women with ovarian cancer and those without.^7^–^10^ However, the methods used to identify potential symptoms have been limited as they have frequently relied on medical records,^8^,^9^ which can underestimate the number and severity of symptoms. Other studies have collected data retrospectively directly from women.^6^,^11^ However, as the 5-year prognosis of ovarian cancer is poor, this may introduce survivor bias. Additionally, in several cases, symptom data have been recorded using checklists, which have been limited in the range of symptoms.^6^,^10^–^17^

This paper reports the results of a study that used both qualitative and quantitative methods to investigate symptoms associated with ovarian cancer.

## Methods

Women urgently referred with a suspicion of ovarian cancer or recently diagnosed with ovarian cancer were recruited from hospital clinics. In order to reduce recall and survivor biases, participants were interviewed prior to diagnosis. However, this was not possible in all cases (e.g. women who had been undergoing investigations in other hospital departments); therefore, some women were interviewed shortly after a diagnosis was made. The method of diagnosis (radiological imaging or histopathology) was independent of the study and did not influence the timing of the interview.

Potential participants were provided with a study information package on arrival at the hospital following the GP referral. Researchers contacted those who returned an initial consent form to arrange a convenient time for the interview. Due to the pragmatic way in which recruitment packs were distributed to women, complete data on nonresponders are not available. A final consent form was completed at the interview. No women withdrew from the study at the time of the interview.

Final diagnoses were obtained from hospital records.

Semistructured interviews were tape recorded and transcribed verbatim. A thematic analysis of the interview data was conducted. This involved obtaining a detailed knowledge of the content of the interview transcripts through in-depth reading and consideration of the text. A thematic framework was developed by identifying key issues within the data (using *a priori* issues and questions from the aims of the study in addition to issues raised by the participants). Passages of text were coded according to each issue or theme identified, and the data were subsequently arranged by each issue or theme.^18^ The qualitative software package ATLAS-ti (Scientific Software Development, Berlin, Germany)^19^ was used. Symptom experiences for women diagnosed with and without ovarian cancer were compared.

Formal sample size calculations for the quantitative analysis were not performed at the outset of the study because a final decision on the number of variables to be included in the regression model could not be made a priori. However, Altman suggests that the number of variables to be considered should be restricted to one-tenth of the sample size, and the maximum size of a model to be the square root of the sample size.^20^ By this rule, including data for 120 women in the preliminary analysis would allow 12 variables to enter the model, with 10 or 11 explanatory variables in the final model.

Following the thematic analysis, the data from the interviews with women were transformed from qualitative data into quantitative data. An assessment was made of the symptoms experienced by each individual (present or absent status was recorded for each symptom, which emerged from the qualitative analysis). This coding was based on the descriptions of the symptoms that women used.

Data were treated as that from a prospective cohort study of symptoms (with follow up continuing until a diagnosis was obtained). The symptom profiles were analysed to identify likely discriminatory features indicative of ovarian cancer using SPSS (SPSS Inc., Chicago, IL, USA)^21^ and Stata (StataCorp LP, College Station, TX, USA).^22^

First, univariate associations were explored between each of the symptom variables and the dependent outcome of ovarian cancer. Statistical significance was assessed using the chi-squared test or Fisher's exact test. The Mann–Whitney *U* test was used to explore the distribution of age with outcome group. Symptoms that reached a significance level of 0.05 (and had greater than five expected numbers) were considered for multivariate analysis using forward stepwise regression. Other potential confounding symptoms such as vaginal bleeding were also included in the model. The significance level for entry into the model was *P* = 0.05 and for the criteria for removal from the model was *P* = 0.1.

Crude and adjusted odds ratios were calculated for the variables that remained in the final multivariate model.

The London MREC granted ethical approval (MREC/02/2/95).

## Results

Interviews were conducted with 124 women. Final diagnoses comprised 44 malignancies, 59 benign gynaecological pathologies and 21 normal findings. Of the 44 malignancies, 40 were ovarian primaries, 2 were peritoneal primaries and 2 were gynaecological cancers, primary site unknown. The four women diagnosed with peritoneal and genital organ (not otherwise specified) cancers were included with the ovarian cancer cases as the occurrence of such tumours can be clinically and histologically indistinguishable from ovarian cancer.^23^ Twenty-five women had stage III or more advanced disease. The average age of the group of women with cancer was 59 years, with the benign/normal cohort having a significantly younger age of 48. Demographic details are provided in Table 1.

**Table 1:** Demographic details of the 124 eligible women

	Women with cancer (*n* = 44)	Women without cancer (*n* = 80)
**Age (years)**		
<20	2	0
20–29	2	4
30–39	4	14
40–49	7	29
50–59	7	17
60–69	14	7
70+	8	9
**Median age**	59	48
**Country of birth**		
UK	42	65
Non-UK	2	15
**Marital status**		
Married/living as married	28	53
Divorced/separated/widowed	11	14
Single	5	11
Missing data	0	2
**Children**		
Yes	32	57
No	10	20
Missing data	2	3
**Timing of interview**		
Prediagnosis	26	37
Postdiagnosis	18	43
**Mode of referral**		
Urgent to gynae	11	22*
Directly to ultrasound scan	11	58**
Urgent to other specialties	9	
Routine to gynae	4	
Routine to ultrasound scan	1	
Accident & Emergency	5	
Other	3	
**Outcome**	Borderline: 13	Benign ovarian cyst(s): 32
	Stage I: 5	Benign ovarian cyst(s) and fibroids: 7
	Stage II: 1	
	Stage III: 12	Fibroids: 15
	Stage IV: 1	Nothing abnormal detected 21
	Not known:*** 8	Others: 5
	Peritoneal cancer: 2	
	Genital organ cancer not otherwise specified 2	

* 19 of 22 urgent referrals to gynae clinics underwent diagnostic surgery.

** 25 of 58 referred to USS underwent surgery.

*** All women with unstaged carcinoma were receiving chemotherapy or palliative care due to suspected advanced-stage disease (stage III or IV). The stage is not known as the tumour had not been excised and staged prior to treatment commencing.

Approximately 60% of the women with cancer were interviewed before diagnosis (26/44), and 46% (37/80) of the noncancer group were interviewed prior to diagnosis.

### Qualitative results

All women diagnosed with ovarian cancer experienced symptoms before diagnosis demonstrating that ovarian cancer is not a silent killer. However, these events were not interpreted as warning signs or symptoms—women saw them as normal changes attributable to ageing, weight gain or other natural physiological processes. This lack of recognition contributed to a delay in seeking medical attention; the median duration of symptoms before interview or diagnosis (whichever occurred first) was 12 months. The symptoms encountered included abdominal pain, distension, postmenopausal bleeding (PMB), fatigue, nausea, vomiting, altered bowel and urinary function, loss of appetite and others.^24^

The terminology used by women to name their symptoms did not always accurately describe the symptoms they experienced. The case of persistent abdominal distension and fluctuating distension/discomfort (both of which women frequently called ‘bloating’) is a striking example. Initially, the women seemed to indicate that they were experiencing a temporary, fluctuating sensation of enlargement or discomfort. When questioned in detail about how they were affected by this symptom, it became apparent that many were experiencing persistent abdominal distension. As a result, during the analysis of the interview transcripts, the coding of bloating and distension was based on women's experiences of these symptoms and not on the terminology used to describe them.

The following passage illustrates how the term ‘bloat’ was used by a woman to describe persistent change (this was coded as distension by the researchers).

Ov 29, 60-69

I’m a size 14 and I went and bought a size 20 skirt last week and it's not big enough. I put it on but I could only stand it for half an hour, even my knickers are leaving a big red line all round me. It is so, you’ve no idea how uncomfortable it its, it's just so up, you feel like you want to stick a pin in it and let loads of air out you know—really bloaty

It is worth noting that distension could occur with or without concomitant bloating.

### Quantitative results

During the transformation process, symptoms were marked as present or absent on the basis of women's descriptions of their experiences. Abdominal distension and bloating were coded as one variable with three categories: neither bloating nor distension, bloating alone and abdominal distension, with or without bloating. The referent category was neither bloating nor distension.

#### Univariate results

Nine variables were significant at the significance level of *P* < 0.05 and had greater than five expected number of events. As mentioned earlier, the cancer group were significantly older than the noncancer group, which reflects the pattern of incidence of ovarian cancer and the imbalance is due to the prospective nature of the data collection. Of the other eight variables, seven occurred more frequently in the cancer group than in the noncancer group (abdominal distension, early satiety, indigestion, vomiting, loss of appetite, feeling hotter than usual and progression or worsening of symptoms). Intramenstrual bleeding was more prevalent among the noncancer group.

Examination of the variable of abdominal distension and bloating revealed that 38 of the women diagnosed with cancer experienced distension (sensitivity of 86.4%, 95% CI 72.6–94.8%) compared with 38 (47.5%) of those without cancer (specificity of 52.5%, 95% CI 41.0–63.8%). In contrast, only two women with cancer experienced abdominal bloating (without distension) (sensitivity of 4.5%, 95% CI 0.6–15.5%) compared with 22 (27.5%) women without cancer (specificity of 72.5%, 95% CI 61.4–81.9%). This pattern has not previously been reported.

#### Multivariate results

The nine variables above (including age) and those of abnormal vaginal bleeding (as likely to be affected by the age difference between the two groups) were entered into the main effects model, resulting in 15 variables being included. The six variables associated with abnormal vaginal bleeding were menorrhagia, missed or irregular periods, PMB, postcoital bleeding, vaginal discharge and worsening of other symptoms while experiencing bleeding.

Multivariate analysis revealed abdominal distension, PMB, loss of appetite, early satiety and progressive symptoms as independent variables associated with ovarian cancer (Table 2). Bloating was not associated with ovarian malignancy.

**Table 2:** Multivariable logistic regression analysis of prediagnosis symptoms associated with the diagnosis of ovarian cancer

Variable*	Number (%) with this variable present		Odds ratios	
		
	Cases (*n* = 44)	Noncases (*n* = 80)	Crude	Adjusted (95% CI)
**Bloating and distension**				
None	4 (9.1)	22 (27.5)	1.0	1.0
Bloating alone	2 (4.5)	22 (27.5)	0.6	0.4 (0.0–4.1)
Abdominal distension (±bloating)	38 (86.4)	38 (47.5)	5.5	5.2 (1.3–20.5)
**Early satiety**	18 (40.9)	7 (8.8)	7.2	5.0 (1.6–15.7)
**Loss of appetite**	17 (38.6)	13 (16.3)	3.2	3.2 (1.1–9.2)
**PMB**	6 (13.6)	2 (2.5)	6.2	9.2 (1.1–76.1)
**Progression/worsening of symptoms**	26 (59.1)	29 (36.3)	2.5	3.6 (1.3–9.8)

* For all risk measures, ‘none’ is the reference category. The analysis is adjusted for age.

A premature feeling of fullness while eating (early satiety) was also strongly associated with the presence of ovarian cancer and, like persistent distension, presumably reflects the presence of an abdominal or pelvic mass. These are both uncommon symptoms in primary care. Although the estimated likelihood ratios are modest (persistent distension 1.4; early satiety 4.7), many of the women with these symptoms who did not have cancer had a nonmalignant mass such as an ovarian cyst or fibroids (26 of 38 with distension and 7 of 7 with early satiety).

The discriminatory power of the model was 81.5% (66% of ovarian cancer cases were correctly classified and 90% of noncases).

## Discussion

Ovarian cancer is not a silent killer. All women with ovarian cancer experienced symptoms prior to diagnosis. While this is not surprising, as the cohort of women in the study had consulted their GPs and been referred to hospital, our findings do suggest that the symptom experiences of women ultimately diagnosed with ovarian cancer are different from those not diagnosed with ovarian malignancy. This has important implications as the majority of women currently diagnosed with ovarian cancer are not initially referred to gynaecological cancer clinics.^5^ Moreover, the symptoms appear to have been present for some time (median 12 months) prior to diagnosis. Similarly, a recent consensus statement,^25^ which was accompanied by an editorial in the *Lancet*,^26^ concluded that women do have symptoms, primarily gastrointestinal and urinary, for several months prior to diagnosis.

Qualitative and quantitative analyses demonstrated that persistent abdominal distension was associated with the diagnosis of ovarian cancer (38 of 44 women with cancer). In contrast, bloating (fluctuating change) was not shown to be associated with the disease (2 of 44 with cancer). This is in opposition to previous research, which has reported that abdominal bloating is one of the main features of ovarian cancer.^12^,^14^,^16^ The discrepancy may be attributed to the novel approach to data collection used in this study—the use of qualitative and quantitative methods enabled the identification of a much broader set of potential symptoms and allowed a deeper understanding of women's symptom experiences.

The terminology used by women to name their symptoms did not always accurately describe the symptoms they experienced. This was most evident in the case of persistent abdominal distension and fluctuating distension/discomfort, both of which women frequently called bloating. Such duality of labelling by the women was an unexpected finding. It is likely that previous research has suffered from over-reporting and misinterpretation of the term bloating.

Women frequently present in primary care with bloating. However, persistent abdominal distension is significantly less common. In order to distinguish between women experiencing persistent abdominal distension and those with fluctuating change, GPs could further question women consulting with symptoms of bloating. Women who might benefit from further investigations and referral to gynaecological cancer clinics may therefore be identified.

Goff *et al.* have recently developed a symptom index for ovarian cancer.^10^ The index was subsequently tested in a separate group of women and controls. Although this technique of development and validation is the preferred method of developing a set of discriminatory features, the data were collected using a symptom checklist. The use of checklists can be problematic because the data collected rely on the checklist being comprehensive and women's interpretations of the listed symptoms are unknown. Previous research (including the Goff study) has tended to group together abdominal distension and bloating into one broad checklist category, thereby limiting opportunities to investigate the subtleties of the distinction we have found. Also, it is possible that women experiencing persistent abdominal distension completing a checklist including a symptom such as bloating would tick that checkbox in addition to the one corresponding to an increase in abdominal size. The occurrence of bloating would therefore be overestimated.

Other previous symptom research has used medical records to collect data, but this is likely to under-represent the experiences of women, as only those symptoms that are deemed salient by the clinician tend to be documented. This was illustrated in a systematic review and meta-analysis, carried out as part of this research: medical record data indicated that 22.6% of women were asymptomatic, whereas only 7.2% of women reporting data directly had no symptoms.^7^

A limitation of our mixed-method approach was that it was necessary to restrict the sample size in order to effectively manage the qualitative data collection and analysis. Although the data allowed the development of a discriminatory model to estimate the magnitude of association between each symptom and a cancer diagnosis, the confidence intervals were wide and the analysis should be considered as hypothesis generating. The model was not tested in an independent data set, and therefore, further validation is required. However, given the limitations of previous research (largely retrospective, use of medical notes, use of restrictive symptom checklists for direct data collection), the present study has made important progress in identifying symptoms that may be indicative of ovarian cancer. Although the predictive values need validation, the requirement to listen to symptom narratives with care and explore what women are describing in using the term bloating is clear.

Although every effort was made to interview participants prior to diagnosis, this only occurred in just over half of the participants. However, there did not seem to be any qualitative difference in the symptom profiles of women interviewed in the differing time frames. Furthermore, a subgroup analysis examining the frequencies of symptoms within the cancer group was conducted to investigate if there were any differences between the interviews conducted before diagnosis and those completed after diagnosis. If a bias exists, symptoms should be reported more frequently in the interviews conducted after diagnosis as a greater emphasis may be placed on these symptoms once a participant knows that they have got a significant disease. However, the opposite pattern was observed in the subgroup analysis, and therefore, it is reasonable to conclude that a systematic bias was not introduced due to the timing of the interview.

Nonresponse bias is a possibility, and this would need to be carefully considered in a future validation study. However, no exclusion criteria were used, and therefore, women with poor performance status were included, so we believe that information representing a broad spectrum of the disease profile was obtained.

## Conclusions

In the absence of more definitive diagnostic tools, early detection of ovarian cancer will continue to challenge the skill of astute clinicians as well as their accumulated scientific acumen.^27^ This study has shown that there may be an opportunity to effect a change in primary care if GPs were to probe a little deeper in order to distinguish between persistent and fluctuating distension as this difference has the potential to discriminate between women with and without ovarian cancer, respectively. This simple action may lead to more rapid and appropriate referrals for women with suspected ovarian malignancy.

## Contribution to authorship

C.R.B. made substantial contributions to the conception and design of the study, acquisition, analysis and interpretation of data and drafting and revising the manuscript and has given final approval to this version.

C.C. substantially contributed to the acquisition, analysis and interpretation of data and drafting and revising the manuscript and has given final approval to this version.

H.S.-L. substantially contributed to the acquisition, analysis and interpretation of data and drafting and revising the manuscript and has given final approval to this version.

P.R. made substantial contributions to the conception and design of the study, interpretation of data and drafting and revising the manuscript and has given final approval to this version.

S.W. made substantial contributions to the analysis and interpretation of data and drafting and revising the manuscript and has given final approval to this version.

A.C. substantially contributed to the conception and design of the study, analysis and interpretation of data and drafting and revising the manuscript and has given final approval to this version.

D.M. substantially contributed to the conception and design of the study, analysis and interpretation of data and drafting and revising the manuscript and has given final approval to this version.

S.T.K. made substantial contributions to the conception and design of the study, acquisition, analysis and interpretation of data and drafting and revising the manuscript and has given final approval to this version.

J.A. made substantial contributions to the conception and design of the study, acquisition, analysis and interpretation of data and drafting and revising the manuscript and has given final approval to this version.

## Details of ethical approval

Ethical approval was granted in February 2003 by the London MREC (MREC/02/2/95).

## Funding

Funding was provided by the Policy Research Programme at the Department of Health and Cancer Research UK (grant number C73/A2916).
